# miR‐375 Regulates Extracellular Vesicle Secretion From *Giardia duodenalis* via Targeting Rab1a

**DOI:** 10.1155/tbed/8019558

**Published:** 2026-08-03

**Authors:** Shaoxiong Liu, Jianqi Yuan, Yanhui Yu, Qinqin Jin, Jianhua Li, Nan Zhang, Xin Li, Xiaocen Wang, Xu Zhang, Pengtao Gong, Lili Cao

**Affiliations:** ^1^ State Key Laboratory for Diagnosis and Treatment of Severe Zoonotic Infectious Diseases, Key Laboratory for Zoonosis Research of the Ministry of Education, and College of Veterinary Medicine, Jilin University, Changchun 130062, China, jlu.edu.cn; ^2^ Second Affiliated Hospital, Jilin University, Changchun, Jilin, China, jlu.edu.cn; ^3^ Jilin Academy of Animal Husbandry and Veterinary Medicine, Changchun, Jilin, China

**Keywords:** extracellular vesicles, *Giardia duodenalis*, miR-375, Rab1a

## Abstract

*Giardia duodenalis* (*G. duodenalis*) represents a prevalent zoonotic protozoan pathogen responsible for diarrheal disorders across human and animal hosts. To date, no licensed effective vaccines have been developed against giardiasis, and the emergence of drug resistance progressively impairs the efficacy of conventional clinical chemotherapy. Extracellular vesicles (EVs) derived from *G. duodenalis* (GEVs) exert pivotal functions in mediating parasite immune evasion as well as the initiation of host inflammatory cascades; nevertheless, the molecular regulatory circuits underlying GEV biosynthesis and secretion remain poorly defined. Previous bioinformatic predictions implicate Rab1a in vesicular trafficking within *G. duodenalis*, whereas its concrete biological contribution to GEV secretion has not been experimentally validated. The present work was designed to screen microRNAs (miRNAs) targeting Rab1a and further elucidate their modulatory effects on GEVs biogenesis. GEVs were purified from *G. duodenalis* WB‐strain trophozoites, and their identity was verified via transmission electron microscopy (TEM), nanoparticle tracking analysis (NTA, modal diameter: 164.7 nm), plus Western blot detection of classic extracellular vesicle biomarker proteins. High‐throughput miRNA sequencing was subsequently conducted to filter candidate miRNAs capable of interacting with the Rab1a 3′ untranslated region (3′UTR), from which miR‐375, miR‐133, and miR‐999 were shortlisted as prospective regulatory molecules. Functional verification revealed that only miR‐375 robustly repressed Rab1a expression at both mRNA and protein abundances. Dual‐luciferase reporter assays further authenticated the direct physical interaction between miR‐375 and the Rab1a 3′UTR. Consistently, ectopic overexpression of miR‐375 resulted in a prominent decline in cellular GEV secretion. In summary, our experimental data corroborate that endogenous *G. duodenalis* miR‐375 suppresses Rab1a expression at the post‐transcriptional level to constrain downstream GEV release. The newly characterized miR‐375‐Rab1a‐GEVs regulatory axis deepens our mechanistic understanding of post‐transcriptional modulation governing vesicle formation in *G. duodenalis*.

## 1. Introduction

*G. duodenalis* is a binucleated flagellated protozoan consisting of two discrete developmental stages: motile trophozoites and environmentally resilient dormant cysts, and it is categorized among the earliest‐diverging extant eukaryotes. Over 95% of its whole‐genome sequence has been deciphered, rendering this organism an ideal experimental model for cellular differentiation research [1]. Multilocus genotyping has classified global *G. duodenalis* isolates into eight distinct assemblages (A–H), among which assemblages A and B are predominantly human with well‐documented zoonotic spillover events [2, 3]. While the majority of giardiasis infections remain clinically asymptomatic, a notable subset of infected individuals develops abdominal distension, watery diarrhea, and nutrient malabsorption; persistent chronic diarrhea in pediatric patients further causes irreversible growth retardation [4, 5]. Notably, immunocompromised populations, including HIV/AIDS sufferers and patients undergoing clinical chemotherapy, display a 1.7–3.8‐fold higher infection prevalence relative to immunocompetent healthy cohorts [6, 7]. Globally, the prevalence of human giardiasis varies extensively from 0.9% to 48.1%, with the highest disease burden concentrated in underresourced territories across Latin America, Africa, and Asia. Domestic livestock such as cattle and sheep, alongside companion dogs and cats, carry parasite carriage rates ranging from 13.6% to 28.0%, whereas the carriage proportion in wild animal populations peaks at ~19%; fecally contaminated drinking water and substandard sanitary conditions constitute the core transmission contributors [8, 9]. Given the poorly characterized interplay among parasite genotype, virulence phenotype, and host susceptibility, unraveling the core molecular cascades regulating parasite differentiation and host immune modulation is critical to pinpoint actionable therapeutic targets for giardiasis intervention.

During host colonization, *G. duodenalis* secretes abundant parasite‐derived extracellular vesicles (EVs) termed GEVs, which feature characteristic cup‐shaped morphology and an average diameter of ~150 nm, with their cargo enriched in diverse proteins, lipids, and functional RNAs readily internalized by recipient host cells [10–13]. Accumulating functional evidence demonstrates that GEVs trigger downstream activation of TLR2, the NLRP3 inflammasome, and the p38/ERK/NF‐κB signaling cascade, consequently elevating the transcription and secretion of proinflammatory mediators IL‐1β, IL‐6, and TNF‐α to amplify parasite‐initiated innate immune responses [14]. On the contrary, GEVs decorated with variant surface proteins (VSPs) directly interact with host NLRP3, hinder canonical inflammasome assembly, and suppress host cell pyroptosis, ultimately facilitating *Giardia* immune evasion from host defensive clearance [15]. Accordingly, pharmacological inhibition of GEV biogenesis and secretion is hypothesized to mitigate *Giardia*‐induced pathological injuries. Previous investigations have validated Vps4a and Rab11 as positive modulators governing GEV production, and functional blockade of either factor effectively abolishes GEV release. Rab1 belongs to the highly conserved Ras‐superfamily small GTPases, which dynamically toggle between GTP‐bound active and GDP‐bound inactive conformations to coordinate bidirectional anterograde and retrograde vesicular trafficking between the endoplasmic reticulum (ER) and the Golgi complex. Nevertheless, it remains experimentally unconfirmed whether Rab1 participates in GEV secretion in *G. duodenalis*.

MicroRNAs (miRNAs) represent a class of 19–25‐nucleotide noncoding RNAs that commonly bind to the 3′‐ or 5′‐untranslated region (UTR) regions of target messenger RNAs to suppress translational initiation or accelerate target mRNA degradation, thereby orchestrating multiple fundamental cellular events, including cell proliferation and apoptosis [16]. Despite its phylogenetically basal evolutionary status, *Giardia* retains an intact and functionally competent miRNA biogenesis machinery. Endogenous Dicer and Argonaute orthologues in this protozoan process precursor molecules derived from snoRNAs or open reading frames to generate mature miRNAs, including miR‐2, miR‐4, miR‐5, miR‐6, and miR‐10; these mature miRNAs predominantly exert post‐transcriptional repression via translational inhibition rather than target mRNA cleavage to fine‐tune the expression of essential effector genes such as VSP‐coding transcripts [17–20]. In silico genome‐wide target prediction further reveals that miR‐5 and miR‐6 potentially target 44–159 distinct VSP genes, indicative of their widespread regulatory roles in modulating transcripts associated with cellular membrane trafficking [19].

In the present work, we prioritized *G. duodenalis* Rab1a as the core target effector protein. High‐throughput miRNA sequencing performed on trophozoites of the *G. duodenalis* WB strain identified miR‐375 as a robust candidate miRNA capable of directly targeting Rab1a. Exogenous introduction of synthetic miR‐375 mimics via parasite electroporation markedly downregulated endogenous Rab1a abundance at both mRNA and protein levels and simultaneously impaired cellular GEV secretion. Subsequent dual‐luciferase reporter mapping verified a single functional seed‐binding motif within the Rab1a 3′‐UTR responsible for miR‐375‐mediated repression, which delineates a previously unappreciated post‐transcriptional regulatory axis controlling GEV release. Collectively, our findings uncover the regulatory role of miR‐375 in governing GEV secretion and lay a mechanistic framework for further basic research focusing on the host–parasite interaction of giardiasis.

## 2. Materials and Methods

### 2.1. Parasite and Cell Culture

*G. duodenalis* WB isolate (ATCC 30957) trophozoites were routinely cultured at 37°C in modified TYI‐S‐33 complete medium supplemented with 12.5% (v/v) heat‐inactivated fetal bovine serum (Every Green, Zhejiang, China), 0.1% (w/v) bovine bile (Sigma–Aldrich, USA), 50 µg mL^−1^ gentamicin sulfate, 100 U mL^−1^ penicillin, and 100 U mL^−1^ streptomycin (Biological Industries, Israel). For parasite collection, intact trophozoite monolayers were prechilled on ice for 20 min to induce detachment, followed by centrifugation at 400 ×*g* for 8 min at 4°C to harvest cell pellets.

HEK293T cells (ATCC CRL‐3216) were maintained in high‐glucose DMEM (Gibco #11995065) supplemented with 10% (v/v) FBS (Gibco), 2.05 mM L‐glutamine, 1 mM sodium pyruvate, 100 U mL^−1^ penicillin, and 100 μg mL^−1^ streptomycin. Cell incubation was performed at 37°C in a humidified incubator supplied with 5% CO_2_. For routine subculturing, adherent cell monolayers were washed twice with precooled PBS, digested with 0.25% trypsin‐EDTA solution at 37°C for 2–3 min, and digestion was terminated by the addition of complete growth medium. Cell suspensions were pelleted via centrifugation at 300 ×*g* for 3 min and reseeded at a 1:4 split ratio for subsequent passage.

### 2.2. Preparation of GEVs Derived From *G. duodenalis* WB Trophozoites

A total of 2 × 10^8^ midlogarithmic‐phase WB trophozoites were cultured, after which the complete culture medium was replaced with serum‐free TYI‐S‐33 medium and cells were further incubated for 24 h. The conditioned culture supernatant was harvested and subjected to sequential differential centrifugation: first centrifugation at 2000 rpm for 10 min at ambient temperature to eliminate intact trophozoites, followed by secondary centrifugation of the retained supernatant at 8000 rpm for 1 h at 4°C. The clarified supernatant was filtered through a 0.22 µm syringe filter prior to ultracentrifugation at 120 000 ×*g* for 1 h at 4°C. The resultant crude GEVs pellet was rinsed twice using 1 mL sterile PBS under identical ultracentrifugation parameters, and the final purified GEVs precipitate was resuspended in 50 µL sterile PBS for downstream experiments.

### 2.3. Plasmid Construction

Total genomic DNA was extracted from cultured *G. duodenalis* trophozoites using a commercial genomic DNA extraction kit (TIANGEN, Cat. DP304‐02, Beijing, China) in strict accordance with the manufacturer’s recommended protocols. Purified genomic DNA served as the template for target gene amplification via PCR, with the unified thermal cycling program set as follows: initial denaturation at 94°C for 5 min; 34 amplification cycles consisting of 95°C denaturation for 30 s, 55°C annealing for 60 s, and 72°C extension for 2 min; and a final prolonged extension step at 72°C for 10 min. Using this standardized PCR protocol, full open reading frames of *G. duodenalis* Rab1a, 14‐3‐3, PDI2, V‐SNARE, and TrxR were successfully amplified. Recovered PCR amplicons were digested with corresponding restriction endonucleases and ligated into the restriction‐digested empty pET‐28a backbone to generate recombinant plasmids pET‐28a‐Rab1a, pET‐28a‐14‐3‐3, pET‐28a‐PDI2, pET‐28a‐V‐SNARE, and pET‐28a‐TrxR; detailed construct information is summarized in Table 1.

**Table 1:** Primers for constructing prokaryotic expression vector plasmids.

Gene	Genebank number	Primer sequences (5′ to 3′)	Cloning site	Primer length (nt)	Product size (bp)
Rab1a	XM_001704374.1	CGC*GGATCC*ATGGCCAGTCCTAATCACG	*Bam*HI	28	639
		CCG*GAATTC*TCAGCAGCACCCCTTG	*Eco*RI	25	
14‐3‐3	XM_001706703.1	CGC*GGATCC*ATGGCCGAGGCATTTACG	*Bam*HI	27	747
		CCG*CTCGAG*TCTTCTCCTCGGCATTATCGTC	*Xho*I	31	
PDI2	XM_001707730.1	CGC*GGATCC*ATGGTTCTTGGACTTCTCTG	*Bam*HI	29	1350
		CCG*CTCGAG*TCTTCTTGCGCTTCTC	*Xho*I	25	
SNARE	XM_001709707.2	CGC*GGATCC*ATGCTTCCTGTCG	*Bam*HI	22	717
		CCG*GAATTC*TTACCTGAAGAAAATC	*Eco*RI	25	
TrxR	XM_001707116.1	CGC*GGATCC*ATGTCCACTCAGCGCC	*Bam*HI	25	945
		CCG*CTCGAG*TCTCCTGCATGGCAAGCC	*Xho*I	27	

Based on the annotated Rab1a transcript GL50803_009558 retrieved from the *Giardia*DB database (https://giardiadb.org/giardiadb/app), the native Rab1a 3′‐UTR sequence is as follows:

5´‐TCTAGATTGATAATATGGTGCCTCGAAATAAATTGTTCATTGGTCGAC‐3´ was chemically synthesized and inserted between the *XbaI* and *SalI* restriction sites of the pmir‐GLO dual‐luciferase reporter vector to produce pmir‐GLO‐Rab1a 3′UTR. To verify the predicted miR‐375 binding site, the seed‐targeting core region was site‐directed mutated into 5´‐TCTAGATTGATAATATGGTGCCTGCTTATTTAGGTGGGTAAGGTCGAC‐3´. The mutated DNA fragment was subcloned into the identical pmir‐GLO backbone to obtain the mutant reporter plasmid pmir‐GLO‐Rab1a 3′UTR mutant.

### 2.4. Preparation of Polyclonal Antibodies Against *Giardia* Rab1a, 14‐3‐3, V‐SNARE, PDI2, and TrxR

Recombinant prokaryotic plasmids pET‐28a‐Rab1a, pET‐28a‐14‐3‐3, pET‐28a‐V‐SNARE, pET‐28a‐PDI2, and pET‐28a‐TrxR were individually transformed into competent *Escherichia coli* BL21 cells. Sequence‐verified positive transformants were cultured in 600 mL of LB medium at 37°C. Once the optical density at 600 nm (OD_600_) reached 0.4–0.8, recombinant protein expression was induced by supplementation with 0.1% (v/v) 100 mM IPTG, followed by an additional 8 h incubation. Bacterial pellets were harvested via centrifugation at 8000 rpm for 15 min at 4°C; after discarding supernatants, cell precipitates were rinsed once with preprepared binding buffer and resuspended in 80 mL of the same buffer. Cell lysis was achieved by sonication set at 85% output power with an alternating 5 s on/5 s off cycle for a total duration of 2 h. The resultant lysate was clarified by centrifugation at 8000 rpm for 15 min at 4°C, and the retained supernatant was loaded onto a gravity chromatography column prepacked with Ni‐NTA Beads 6FF (Solarbio, China; Cat. Number P2010). The column matrix was washed with 10 column volumes of wash buffer, and target recombinant proteins were subsequently eluted using 10 mL of elution buffer.

For antibody production, male SPF‐grade New Zealand white rabbits (2 months old, body weight: 2.0 ± 0.2 kg, Liaoning Changsheng Biotechnology Co., Ltd., Benxi, China) received subcutaneous immunization. The primary immunization was performed with 500 µg purified individual protein emulsified at a 1:1 (v/v) ratio with complete Freund’s adjuvant (Sigma‐Aldrich, USA; Cat. Number F5881). A booster injection containing 500 µg identical protein mixed 1:1 (v/v) with incomplete Freund’s adjuvant (Sigma‐Aldrich, USA; Cat. Number F5506) was administered 14 days postprimary immunization, and a third equivalent booster was supplemented at day 7 after the second injection. Whole blood was collected 7 days following the final booster and kept at 4°C overnight to allow complete clot formation. Serum fractions were separated by centrifugation at 2000 ×*g* for 10 min at 4°C, split into small aliquots, and preserved at −80°C for subsequent use.

### 2.5. Dual‐Luciferase Reporter Assay

HEK293T cells were seeded into 6‐well culture plates and incubated for 24 h prior to transient transfection. For experimental groups: Group A was cotransfected with 1 µg pmir‐GLO‐Rab1a‐3′UTR plasmid plus 80 pM miR‐375 mimics (sequence: 5′‐AGTACGCTCGGCTTGCTTGTTT‐3′; working concentration recommended by the reagent manufacturer); Group B received 1 µg pmir‐GLO‐Rab1a‐3′UTR‐mutant together with an equal dose of 80 pM miR‐375 mimics. Two blank control groups were set correspondingly: C1 transfected with pmir‐GLO‐Rab1a‐3′UTR alone and C2 with pmir‐GLO‐Rab1a‐3′UTR‐mutant only.

For each transfection setup, plasmid DNA was diluted in 200 µL Opti‐MEM medium (Gibco, USA; Cat. Number 31985‐070), while 8 µL Lipofectamine 2000 transfection reagent (Invitrogen, USA; Cat. Number 11668‐019) was separately diluted in another 200 µL Opti‐MEM. After 15 min of incubation at ambient temperature to form DNA‐lipid complexes, the mixed solution was gently added to adherent cells. Following 6 h of cultivation at 37°C with 5% CO_2_, the initial medium was replaced with complete high‐glucose DMEM supplemented with 10% FBS and 1% penicillin–streptomycin. Cells were harvested at 24 h post‐transfection, and dual‐luciferase luminescence values were quantified using the Dual‐Luciferase Reporter Assay Kit (Yeasen, China; Cat. Number 11402ES60) in strict accordance with the manufacturer’s operational specifications.

### 2.6. Transmission Electron Microscopy (TEM)

For negative‐staining TEM observation, a 10 µL aliquot of purified GEV suspension was dropped onto carbon‐coated copper grids and incubated for 1 min at room temperature to allow adequate sample adhesion. Subsequently, 20 µL of 3% (w/v) phosphotungstic acid was applied for 5 min for negative contrast staining. The Formvar‐supported GEV samples were visualized using a HITACHI HT‐7800 transmission electron microscope with an operating voltage of 120 kV.

### 2.7. Nanoparticle Tracking Analysis (NTA)

The particle size distribution and concentration of isolated GEVs were quantified via NTA using a ZetaView PMX 110 system (Particle Metrix, Germany). Briefly, freshly prepared GEVs were resuspended in sterile PBS to a final volume of 1 mL and serially diluted to achieve an optimal concentration of 30–100 particles per frame (~50 frames^−1^). Five consecutive 60‐s video recordings were captured under standardized instrumental parameters, including a constant chamber temperature of 25°C and an infusion rate of 22 µL s^−1^ (100 arbitrary units). Image acquisition and subsequent data analysis were performed using integrated ZetaView 8.02.28 software, which automatically calculated the mean diameter, modal particle size, and particle concentration (particles mL^−1^) of GEVs.

### 2.8. Statistical Analysis

All statistical analyses were performed using unpaired Student’s *t*‐tests (one‐tailed or two‐tailed according to experimental design), one‐way ANOVA, or two‐way ANOVA. Tukey’s multiple comparison test was applied for post‐hoc analysis after two‐way ANOVA. All quantitative data are presented as the mean ± standard error of the mean (SEM). Statistical significance was defined as  ^∗^ *p* ≤ 0.05,  ^∗∗^ *p* ≤ 0.01, and  ^∗∗∗^ *p* ≤ 0.001. All statistical calculations were conducted using GraphPad Prism software, and all experiments were repeated at least three independent times with consistent and reproducible results.

## 3. Result

### 3.1. Purification And Characterization of GEVs Derived From *G. duodenalis* WB Trophozoites

**Figure 1:**
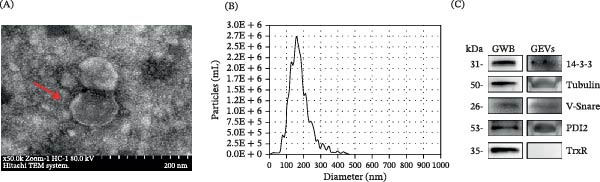
Characterization of *Giardia* extracellular vesicles (GEVs). (A) Representative transmission electron microscopy (TEM) micrograph displaying intact, well‐defined membrane boundaries of purified GEVs. (B) Particle size distribution curve of purified GEVs quantified via nanoparticle tracking analysis (NTA). (C) Western blot‐based identification of canonical biomarker proteins for isolated GEVs.

TEM revealed delineated GEVs ~ 150 nm in diameter (Figure 1A). NTA further determined that the isolated GEVs exhibited a modal diameter of 164.7 nm and a particle concentration of 1.2 × 10^11^ particles mL^−1^ (Figure 1B).

To verify the identity and purity of purified GEVs, rabbit polyclonal antibodies against GEVs‐enriched proteins were prepared for subsequent biomarker validation (Supporting Information 1: Figure S1). Western blotting was performed on GEV lysates to characterize canonical EV marker proteins. Immunoblotting results demonstrated abundant expression of representative *Giardia* EV markers, including 14‐3‐3 (31 kDa), α‐tubulin (50 kDa), V‐SNARE (26 kDa), and PDI2 (53 kDa). In contrast, the intracellular contamination marker TrxR (35 kDa) was undetectable in all purified GEV preparations.

The positive detection of vesicle‐specific proteins coupled with the complete absence of cytoplasmic contaminants confirmed that the isolated vesicles were free of host cell debris and possessed high purity. Collectively, these morphological, particle sizing, and biochemical characterizations verify that the purified particles are bona fide GEVs (Figure 1C), providing reliable experimental materials for subsequent functional investigations on miR‐375‐mediated regulatory mechanisms of GEV secretion.

### 3.2. miRNAome Sequencing of *G. duodenalis* WB Strain Trophozoites

**Figure 2:**
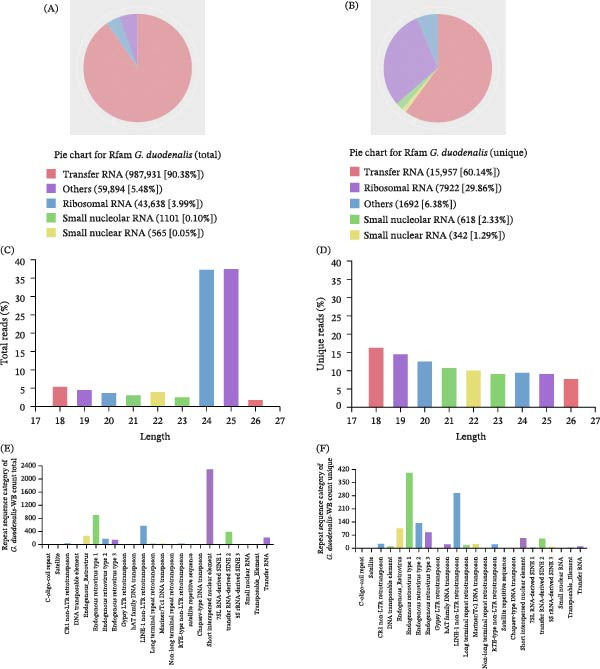
Small RNA and miRNA profiling landscape of *G. duodenalis* WB trophozoites. (A) Relative compositional abundance of distinct RNA biotypes derived from total sequencing reads. (B) Relative proportion of annotated RNA subtypes originating from unique *Giardia*‐genome‐matched reads. (C) Nucleotide length distribution of all detected small RNA/miRNA fragments. (D) Size distribution profile of *Giardia*‐specific miRNA sequences. (E) Classification of genome‐embedded repetitive elements and normalized copy number distribution associated with total mapped miRNAs across the *G. duodenalis* genome. (F) Repeat element grouping and corresponding normalized copy number statistics for *Giardia*‐specific miRNAs.

ACGT101‐miR sequencing (Hangzhou Lianchuan Biotechnology Co., Ltd., Hangzhou, China; Project ID: 2021L16shyB00‐A31) was performed using 2 × 10^8^ mid‐log‐phase *G. duodenalis* WB trophozoites, which generated 1.61 × 10^7^ high‐quality clean reads. Following sequence alignment against the *G. duodenalis* reference genome assembly (UP000001548), mapped RNA sequences were grouped into five annotated biotype categories based on classification criteria from the Rfam database (Figure 2A,). Pie chart quantification revealed that tRNA‐derived small RNAs (tsRNAs) dominated total mapped raw reads, accounting for an overwhelming proportion of 90.38% and representing the most abundant small RNA subset. In contrast, uniquely genome‐aligned reads exhibited distinct compositional patterns, among which fragments originating from snoRNA (2.33%) and snRNA (1.29%) occupied relatively larger fractions.

Beyond canonical Rfam‐annotated noncoding RNAs, abundant short sequencing fragments originating from endogenous tRNA hydrolysis were annotated as tsRNAs. Subsequent length‐distribution analysis (Figure 2C,) demonstrated that these tRNA‐derived fragments were predominantly concentrated at 21–24 nt in both total and unique read pools; this size distribution closely mirrors that of canonical mature miRNAs, whose length peaks at 22 nt (mode ± SD: 22 ± 0.9 nt) and are matured through the conserved Dicer‐dependent cleavage cascade. Such overlapping length signatures suggest that tsRNAs may be subjected to comparable enzymatic maturation and plausibly mediate miRNA‐mimetic regulatory roles within *Giardia*.

Additional repetitive element profiling (Figure 2E,) enabled quantitative mapping of tRNA‐derived repeat sequences across the entire *Giardia* transcriptome. Parallel miRNA screening demonstrated that 68.4% of the total 153 predicted candidate miRNAs reside within or adjacent to retroposon‐associated repetitive loci, with individual genomic copy numbers varying from 1 to 34 per haploid genome. Taken together, robust biogenesis of endogenous tsRNAs accompanied by constitutive canonical miRNA expression strongly supports the existence of an elaborate small RNA‐dominated post‐transcriptional regulatory circuitry in *G. duodenalis*. Full datasets detailing miRNA expression profiles are compiled in Supporting Information 3: Table S1.

### 3.3. Screening and Identification of *Giardia*‐Targeting miRNAs Directed Against Rab1a

**Figure 3:**
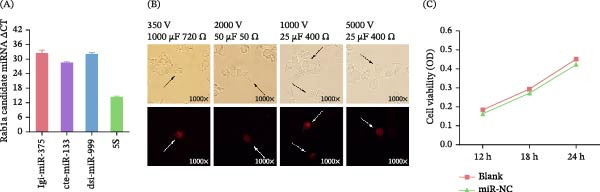
Screening and functional validation of *G. duodenalis* miRNAs targeting Rab1a. (A) qRT‐PCR quantification of endogenous lgi‐miR‐375, cte‐miR‐133, and dsi‐miR‐999 in *G. duodenalis* trophozoites; *G. duodenalis* 5S rRNA was used as the endogenous reference control. (B) Optimization of trophozoite electroporation conditions with Cy3‐labeled miR‐NC serving as a fluorescent reporter. (C) Growth curve analysis evaluating potential cytotoxicity induced by exogenous miRNA electroporation.

Based on the canonical regulatory mechanism whereby miRNA seed sequences bind to the 3′UTR of target mRNAs via perfect or imperfect base pairing, all predicted *G. duodenalis* miRNAs were aligned against the Rab1a 3′UTR. Three candidate miRNAs, including miR‐375, miR‐133, and miR‐999, were preliminarily screened out according to their comprehensive hybridization scores. Subsequent qRT‐PCR validation confirmed high endogenous expression levels of these three miRNAs in *G. duodenalis* trophozoites (Figure 3A).

Electroporation parameters for trophozoite transfection were optimized using Cy3‐labeled miR‐NC as a fluorescent indicator. The strongest intracellular fluorescence signal was detected under electrical conditions of 1000 V, 25 μF, and 400 Ω, which were defined as the optimal electroporation conditions for subsequent experiments (Figure 3B). Under these optimized parameters, transfection with the nontargeting negative control miR‐NC did not affect the proliferative curve of *G. duodenalis* trophozoites, demonstrating that exogenous miRNA transfection causes no obvious cytotoxicity to *G. duodenalis* (Figure 3C).

Furthermore, a prokaryotic expression vector, pET‐28a‐Rab1a, was successfully constructed. The homogeneous recombinant His‐Rab1a protein was obtained via affinity purification and subsequently used for the preparation of polyclonal antiserum in New Zealand white rabbits (Supporting Information 1: Figure S1).

**Figure 4:**
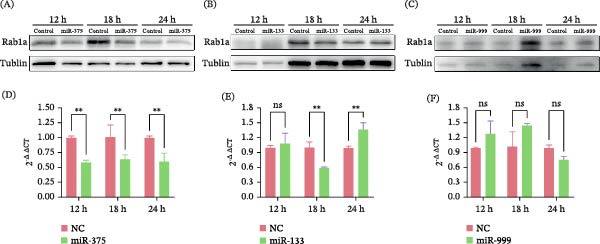
Functional verification of candidate *G. duodenalis* miRNAs capable of targeting Rab1a. (A–C) Western blot analysis of Rab1a (~23 kDa) protein abundance in trophozoites individually transfected with miR‐375, miR‐133 and miR‐999 mimics; antitubulin was used as the internal loading control. (D–F) Parallel qRT‐PCR quantification of relative Rab1a transcript abundance across the corresponding experimental groups. Values are expressed as mean ± SD;  ^∗∗^ *p*  < 0.01 compared with the miR‐NC control group.

Trophozoites of the *G. duodenalis* WB isolates were subjected to electroporation to achieve the overexpression of the three candidate miRNAs. Synthetic mimics corresponding to miR‐375 (Figure 4A), miR‐133 (Figure 4B), and miR‐999 (Figure 4C) were individually transfected into parasites, with parallel transfection of miR‐NC set as the negative blank control. Western blot analysis using a custom anti‐Rab1a antiserum was first implemented to detect endogenous Rab1a protein abundance. An immunoreactive Rab1a band at ~23 kDa was detectable across all experimental groups, whereas pronounced attenuation in band density was exclusively detected in trophozoites receiving miR‐375 mimic treatment.

qRT‐PCR was further conducted to quantify Rab1a transcriptional abundance for independent validation of the above regulatory phenotype. Consistently with the immunoblotting results, Rab1a transcript abundance was reduced by 39.2% in the miR‐375 mimic group relative to the miR‐NC control, reaching statistically significant differences (*p*  < 0.01; Figure 4D–F). Collectively, concordant data from mRNA quantification and immunoprotein detection robustly verify that miR‐375 functions as a post‐transcriptional modulator to negatively regulate Rab1a expression in *G. duodenalis*.

### 3.4. *G. duodenalis* miR‐375 Targeting of Rab1a Dampens GEV Secretion

**Figure 5:**
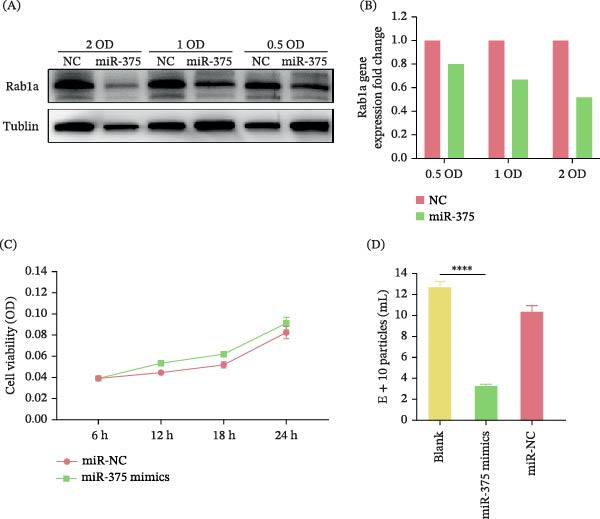
miR‐375 targets Rab1a to dampen GEVs secretion. (A–B) Dose‐dependent suppression of Rab1a protein expression in *G. duodenalis* trophozoites electroporated with gradient concentrations of miR‐375 mimic. (C) Growth curve analysis of *G. duodenalis* trophozoites after transfection with miR‐NC and miR‐375. (D) Nanoparticle tracking analysis (NTA) of GEVs derived from *Giardia* trophozoites transfected with 2 OD miR‐375 mimics.  ^∗∗∗∗^ indicate statistical significance at *p* < 0.0001 (the highest level of significance in our labeling system). Specifically, the significance levels are denoted as  ^∗^ *p* < 0.05,  ^∗∗^ *p* < 0.01,  ^∗∗∗^ *p* < 0.001 and  ^∗∗∗∗^ *p* < 0.0001.

To further characterize the dose‐dependent regulatory effect of miR‐375 on endogenous Rab1a expression, *G. duodenalis* trophozoites were transfected with gradient concentrations of the miR‐375 mimic. Western blot analysis revealed that incremental doses of miR‐375 mimic resulted in a gradual proportional decrease in Rab1a protein abundance, confirming a dose‐dependent inhibitory effect of miR‐375 on Rab1a expression (Figure 5A).

To rule out the possibility that reduced GEVs secretion arises from impaired parasite growth and viability, the proliferative capacity of *G. duodenalis* trophozoites was assessed following miR‐375 overexpression. No significant differences in trophozoite proliferation were observed between the miR‐375 mimic group and the miR‐NC control group (Figure 5C), indicating that miR‐375 exerts no apparent influence on the *G. duodenalis* growth status.

Subsequently, NTA was performed to quantify the yield of secreted GEVs. Consistent with miR‐375‐mediated Rab1a downregulation, NTA results validated that miR‐375 mimic treatment significantly repressed GEV secretion from *G. duodenalis* trophozoites (Supporting Information 2: Figure S2 and Figure 5D). Collectively, these findings demonstrate that miR‐375 suppresses GEVs production via downregulating Rab1a expression in a proliferation‐independent manner in *G. duodenalis*.

**Figure 6:**
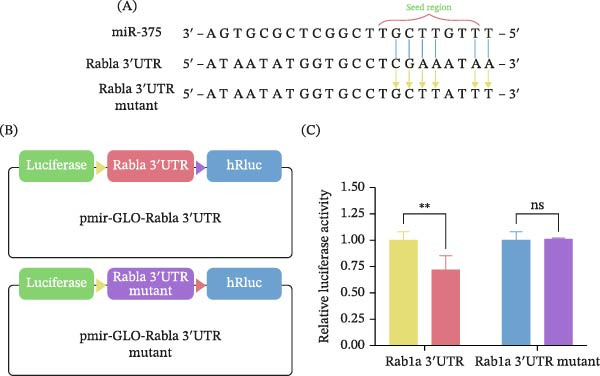
Dual‐luciferase reporter verification of the specific binding between miR‐375 and Rab1a 3^′^‐UTR. (A) In silico prediction of the miR‐375 seed‐match site (CGAAATAA) located within the Rab1a 3^′^‐UTR sequence. (B) Schematic diagram of wild‐type pmir‐GLO‐Rab1a 3^′^UTR and mutant pmir‐GLO‐Rab1a 3^′^UTR luciferase reporter vectors, with the core binding sequence mutated from CGAAATAA to GCTTATTT. (C) Dual‐luciferase assay results confirming the specific targeting relationship between miR‐375 and Rab1a 3^′^UTR. Data are presented as mean ± SD; *p*  < 0.01 versus the miR‐NC group. The  ^∗∗^ indicate statistical significance at *p* < 0.01. Significance labeling system is  ^∗^ *p* < 0.05,  ^∗∗^ *p* < 0.01,  ^∗∗∗^ *p* < 0.001 and  ^∗∗∗∗^ *p* < 0.0001.

In silico prediction analysis identified six putative seed‐matching sites within the 3′UTR of Rab1a. To validate the direct targeting interaction between miR‐375 and Rab1a, the full‐length wild‐type Rab1a 3′UTR and its corresponding mutant fragment carrying the CGAAATAA→GCTTATTT substitution were individually inserted downstream of the firefly luciferase coding sequence in the pmir‐GLO reporter vector (Figure 6A and B). Dual‐luciferase assay results showed that, relative to the miR‐NC group, miR‐375 mimic transfection reduced the firefly/Renilla luciferase activity ratio by ~25% in the wild‐type Rab1a 3′UTR reporter group (*p*  < 0.01). Notably, such an inhibitory effect was completely abrogated in the mutant Rab1a 3′UTR reporter group (Figure 6C). Collectively, these data confirm that the CGAAATAA sequence serves as the core functional binding site for miR‐375, which mediates the post‐transcriptional repression of Rab1a and ultimately reduces GEV secretion.

## 4. Discussion

The present study demonstrates that *G. duodenalis* miR‐375 specifically targets the 3′UTR of Rab1a mRNA, facilitates Rab1a mRNA degradation, reduces Rab1a protein abundance, and ultimately diminishes the secretion of GEVs. These findings not only identify Rab1a as a previously uncharacterized component of the GEV biogenesis machinery but also establish miR‐375 as the first endogenous miRNA capable of modulating GEV export in *G. duodenalis*.

Currently, no licensed vaccines are available for giardiasis, and the widespread emergence of metronidazole resistance has become a pressing clinical concern, emphasizing the necessity of deciphering the molecular crosstalk between *Giardia* and host cells [21]. As an extracellular protozoan pathogen, *Giardia* secretes soluble and vesicular effector molecules to trigger inflammation, pyroptosis, and apoptosis in macrophages and intestinal epithelial cells. EVs are evolutionarily conserved nanoscale vesicles secreted by nearly all cell types, which mediate intercellular communication via the transfer of proteins, lipids, and nucleic acids [22, 23]. Once regarded as trivial cellular waste products, EVs are now considered promising biomarkers and cell‐free therapeutic tools for tissue regeneration, cancer treatment, and immune modulation [24, 25]. Nevertheless, the clinical translation of EV‐based applications is severely constrained by low production yield, poor structural stability, short in vivo half‐life, and insufficient targeting capacity [26, 27].

EV biogenesis is governed by ESCRT‐dependent and ESCRT‐independent pathways, accompanied by precise selective cargo sorting [28–30]. Conventionally adopted EV isolation techniques include ultracentrifugation, size‐exclusion chromatography, polymer‐based precipitation, and immunoaffinity capture, each possessing inherent technical limitations [31, 32]. Genetic and physicochemical modification strategies have been widely applied to engineer EVs with enhanced stability and targeting specificity [33, 34]. In addition, biomaterial‐based delivery platforms, such as hydrogels, microneedles, and microspheres, effectively improve EV retention, sustained release performance, and overall therapeutic efficacy [35–37].

Parasite‐derived EVs play pivotal roles in enhancing pathogen virulence, horizontally transmitting drug resistance, reprogramming host transcriptional profiles, and facilitating parasite adhesion and colonization [38]. In Leishmania, Leishmania‐derived EVs (LEVs) deliver gp63 and other virulence factors to promote complement evasion and host cell invasion [39]. LEVs polarize macrophages toward an M2 phenotype, fine‐tune host inflammatory responses, and construct an intracellular niche conducive to parasite survival [40]. These vesicles also shuttle LRV1 virions and drug resistance‐associated molecules, exacerbating parasite infectivity and compromising chemotherapy efficacy. Owing to their high immunogenicity, LEVs have emerged as promising noninvasive serodiagnostic antigens [41, 42], while host‐derived EVs represent potential host‐directed immunotherapeutic agents for parasitic infections [43].

EVs secreted by *Trichomonas vaginalis* (TvEVs) act as reversible molecular adhesives to strengthen trophozoite adhesion to urogenital epithelial surfaces, providing the initial mechanical support for successful colonization. In vivo coinoculation assays in male mice increase prostate parasite burden by 4–6‐fold and prolong parasite survival, offering direct in vivo evidence that TvEVs facilitate parasitic persistent infections. TvEV cargoes, including tsRNAs, proteases, and heat‐shock proteins, inhibit NF‐κB signaling and induce IL‐10 expression, thereby remodeling the local immune microenvironment to favor parasite survival. The enrichment of Trichomonas‐specific components in TvEVs provides a valuable reservoir for noninvasive diagnosis, and pharmacological blockade of TvEV secretion or uptake represents a viable anti‐trichomonal therapeutic strategy [44]. Our previous study verified that GEVs can be internalized by intestinal epithelial cells, activate the TLR2–NLRP3 inflammasome, and amplify host innate immune responses against *Giardia* infection [10]. Therefore, targeted interference with GEV secretion serves as a rational and unconventional strategy for giardiasis prevention and control.

Organellar membrane trafficking is essential for maintaining the physiological functions of eukaryotic cells. Rab GTPases are core regulatory proteins that govern vesicle budding, motility, tethering, and fusion through the recruitment of downstream effector proteins. The crosstalk among distinct Rab family members ensures the spatiotemporal accuracy of intracellular vesicle transport, and dysfunctional Rab signaling is closely correlated with immunodeficiencies, malignancies, and neurological disorders [45]. The present findings demonstrate that Rab1a acts as a critical regulator of GEV biogenesis in *Giardia*. Consistent with the well‐characterized role of *Giardia* Rab1 in ER–Golgi vesicular trafficking and endomembrane transport, Rab1a mediates multiple key processes during GEV maturation, including vesicle budding, cargo sorting, and extracellular secretion. As previously reported, *Giardia* Rab1 is functionally conserved and localized specifically to secretory vesicles, confirming its central involvement in vesicle formation [46]. Similarly, Rab11 has been shown to mediate intraluminal vesicle (ILV) formation in peripheral vacuoles (PVs) and modulate the release of exosome‐like vesicles in *Giardia*, participating in membrane trafficking and vesicle maturation [47]. In line with these observations, the current study verifies that Rab1a is indispensable for GEVs secretion, as miR‐375‐mediated Rab1a downregulation significantly reduces GEVs production. Both Rab1a and Rab11 participate in the primitive yet highly efficient secretory pathway of *Giardia* and coordinately regulate ER–PV trafficking and vesicle budding.

MiRNAs function as fine‐tuners of intracellular regulatory networks. By base pairing with the 3′UTR of target transcripts, miRNAs trigger mRNA cleavage or translational repression to execute post‐transcriptional gene regulation [48]. Consistent with our results in *G. duodenalis*, small noncoding RNAs universally modulate vesicle secretion in diverse pathogens. In Salmonella, the small regulatory RNA MicA downregulates the outer membrane protein OmpA, alters the OmpA/OmpC expression ratio, and markedly increases outer membrane vesicle abundance; these vesicles further elicit protective Th1/Th17 immune responses against bacterial infection [49]. The present study extends this regulatory paradigm from prokaryotic sRNAs to eukaryotic parasite miRNAs. Mechanistically, miR‐375 suppresses Rab1a expression via specific 3′‐UTR binding and restricts GEV release, adhering to the conserved principle that small RNAs remodel membrane protein homeostasis to control microbial vesicle biogenesis. Notably, studies regarding miRNA‐mediated EV regulation in eukaryotic pathogens remain scarce. Accordingly, the current work establishes the novel miR‐375/Rab1 a/GEVs regulatory axis in *G. duodenalis* and provides robust experimental evidence to enrich future related research.

Based on this mechanistic premise, we conducted an in silico screening of the *G. duodenalis* miRNA repertoire and identified miR‐375 as a high‐confidence Rab1a‐targeting candidate. Functional validation revealed that sustained miR‐375 overexpression (>12 h) induced significant reductions in Rab1a mRNA levels, accompanied by an ~70% decrease in GEV counts, an inhibitory effect comparable to that of Rab11 knockdown. In contrast, miR‐133 and miR‐999 failed to produce stable suppressive effects on GEV secretion. Subsequent dose–response assays confirmed that increased concentrations of miR‐375 mimic proportionally reduced Rab1a protein abundance, demonstrating the specific and dose‐dependent regulatory activity of miR‐375. Of note, attempts to deplete endogenous miR‐375 using synthetic inhibitors were unsuccessful, most likely due to the absence of conserved enzymatic machinery for exogenous antagomiR processing in *G. duodenalis*. This technical limitation was successfully circumvented via the gradient titration assay applied in this study.

MiR‐375 was initially identified as an islet‐specific microRNA [50] and has been characterized as a pleiotropic post‐transcriptional regulator highly enriched in brain, gastrointestinal, and pancreatic tissues [51]. Beyond its canonical role in maintaining glucose homeostasis, miR‐375 modulates immune cell polarization, inflammatory signaling transduction, and multiple developmental processes, including neurogenesis, osteogenesis, adipogenesis, and pancreatic morphogenesis [52–57]. Dysregulated miR‐375 expression is primarily driven by promoter hypermethylation and competitive endogenous RNA network disorders, rendering it a key effector in autoimmune diseases, chronic inflammation, viral infections, and tumorigenesis [58–60]. Consequently, miR‐375 has been increasingly recognized as a promising noninvasive biomarker and a tractable therapeutic target for multiple pathological conditions [61–64]. The present study expands the functional repertoire of miR‐375 and uncovers its previously unrecognized role in modulating parasite extracellular vesicle biogenesis.

Collectively, our findings propose a distinctive “fight‐parasite‐with‐parasite” paradigm, in which endogenous parasite miRNA is harnessed to inhibit vesicle‐mediated host manipulation. Despite these promising mechanistic insights, the clinical translation of this RNA‐based strategy remains hindered by inherent challenges regarding the stability, delivery efficiency, and immunogenicity of RNA therapeutics [65]. Lipid‐based, polymer‐based, inorganic, and exosome‐derived nanocarriers, which have been extensively optimized for human miRNA drugs, can be further adapted for veterinary applications against giardiasis [66]. The combination of nanocarrier‐encapsulated miR‐375 mimics and low‐dose metronidazole may effectively reduce the parasite burden and restrain the emergence of drug resistance. Future studies will focus on validating the therapeutic efficacy of this combination regimen in murine giardiasis models and optimizing carrier systems that resist gastric degradation to achieve efficient miR‐375 mimic delivery within the small intestinal lumen.

## 5. Conclusions

In summary, this study demonstrates that endogenous *G. duodenalis* miR‐375 specifically binds to the canonical seed‐matching site located within the 3′UTR of Rab1a mRNA, thereby triggering the degradation of Rab1a transcripts in a dose‐dependent manner and ultimately downregulating Rab1a protein expression. This specific miRNA–mRNA interaction enables Rab1a to function as a critical molecular valve governing GEV biogenesis, whose repression substantially attenuates GEV secretion. Collectively, these findings establish the feasibility of exploiting parasite‐encoded miRNAs as a precise molecular tool to disrupt vesicle‐mediated host–pathogen communication and provide a novel research prototype for follow‐up fundamental studies focusing on RNA‐related anti‐*Giardia* research.

## Author Contributions

**Shaoxiong Liu**: methodology, data curation, formal analysis, investigation, writing – original draft. **Jianqi Yuan and Yanhui Yu**: methodology, data curation, formal analysis, investigation. **Qinqin Jin**: methodology, investigation. **Xu Zhang**: validation, software, visualization. **Jianhua Li, Nan Zhang, Xin Li, and Xiaocen Wang**: methodology, formal analysis, conceptualization, funding acquisition, supervision, writing – original draft, writing – review and editing. **Pengtao Gong and Lili Cao**: funding acquisition, supervision, project administration, resources, writing – review and editing.

## Funding

This work was funded by the National Natural Science Foundation of China (Grant 32573393) and the China Wool‐Sheep & Cashmere‐Goat Research System (Grant CARS‐39).

## Disclosure

All authors have read and approved the final manuscript.

## Ethics Statement

All animal experiments were conducted in strict accordance with the Regulations for the Administration of Affairs Concerning Experimental Animals approved by the State Council of the People’s Republic of China (1988.11.1) and the Animal Welfare and Research Ethics Committee of Jilin University (IACUC Permit Number SY202407006).

## Conflicts of Interest

The authors declare no conflicts of interest.

## Supporting Information

Additional supporting information can be found online in the Supporting Information section.

## Supporting information


**Supporting Information 1** Figure S1: Titer evaluation of polyclonal antibodies targeting rabbit‐origin *G. duodenalis* 14‐3‐3, V‐SNARE, PDI2, TrxR and Rab1a.

**Supporting Information 2** Figure S2: NTA quantification of GEVs derived from *G. duodenalis* treated with miR‐375 mimics, negative control miRNA (miR‐NC) and blank control.

**Supporting Information 3** Table S1: All Expressed miRNA of *G. duodenalis*.

## Data Availability

The data are available upon request from the authors.
