# Supplementary figures and images for: miR‐375 Regulates Extracellular Vesicle Secretion From Giardia duodenalis via Targeting Rab1a
Source: Transbound Emerg Dis. 2026 Aug 3;2026:8019558. doi: 10.1155/tbed/8019558 (PMC13430286; doi:10.1155/tbed/8019558)

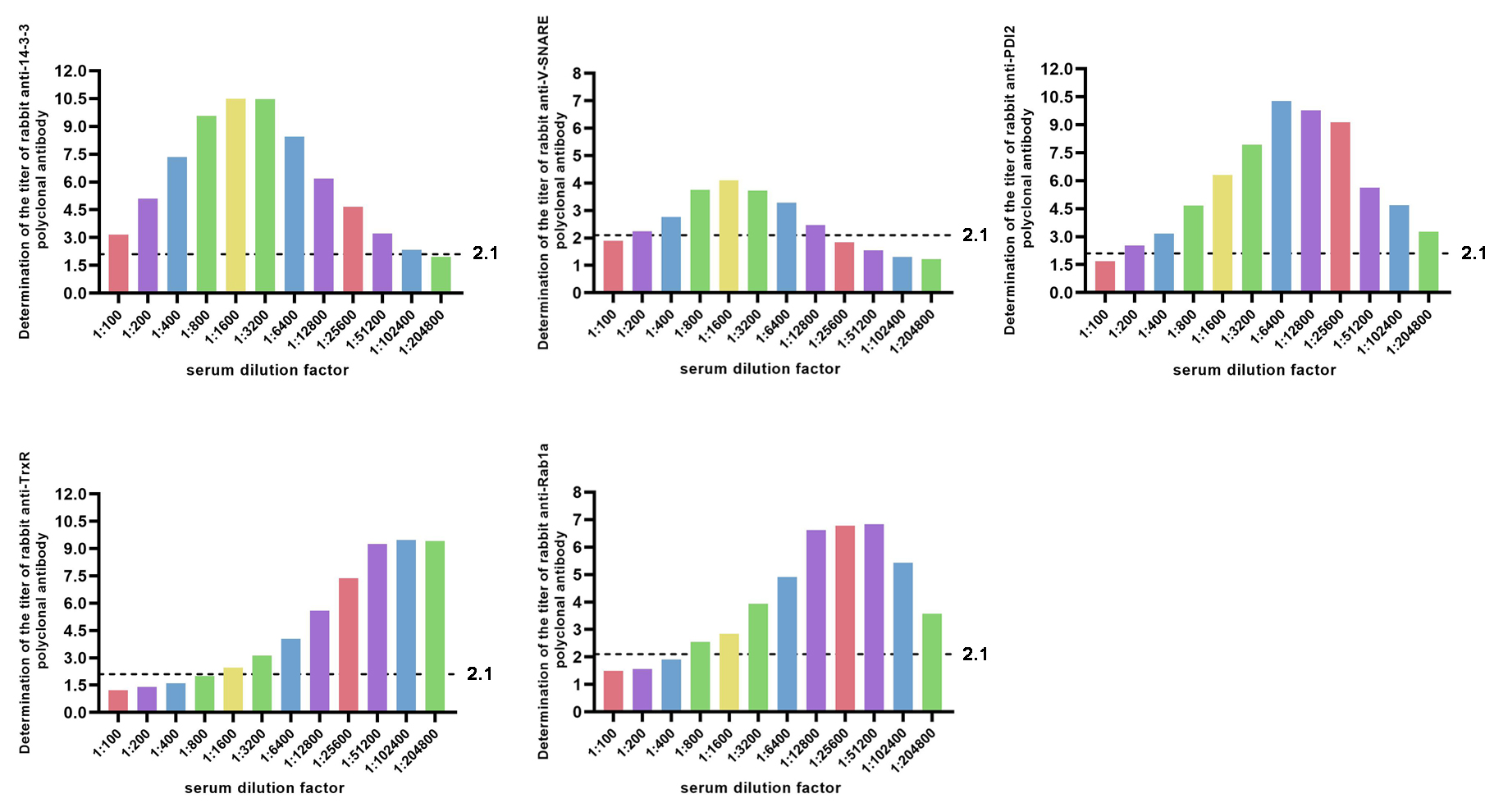

Supplement: Supplementary file 1 — Supporting Information 1 Figure S1: Titer evaluation of polyclonal antibodies targeting rabbit‐origin G. duodenalis 14‐3‐3, V‐SNARE, PDI2, TrxR and Rab1a. [file TBED-2026-8019558-s001.jpg]

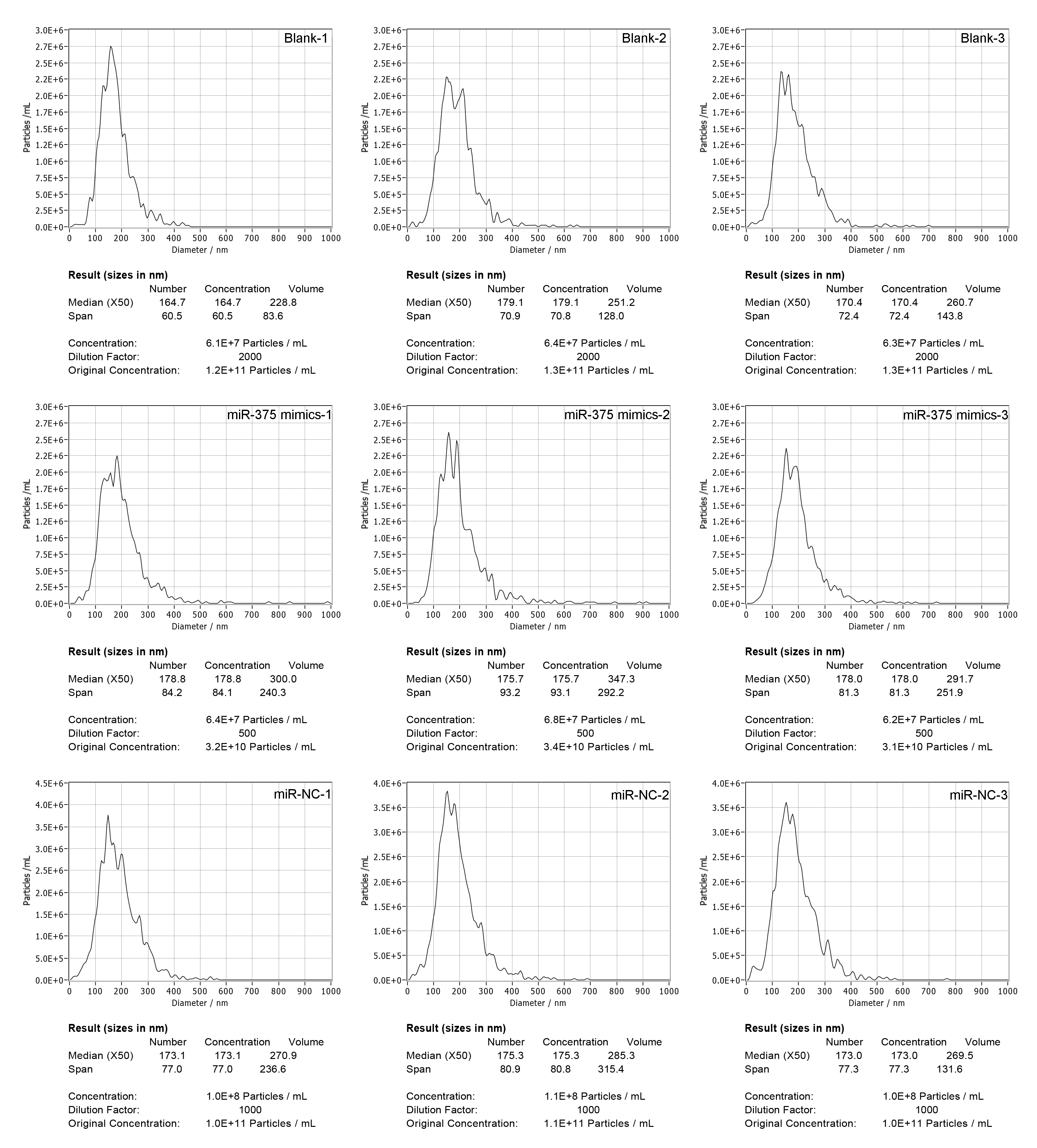

Supplement: Supplementary file 2 — Supporting Information 2 Figure S2: NTA quantification of GEVs derived from G. duodenalis treated with miR‐375 mimics, negative control miRNA (miR‐NC) and blank control. [file TBED-2026-8019558-s002.jpg]
